# ATF3 represses PINK1 gene transcription in lung epithelial cells to control mitochondrial homeostasis

**DOI:** 10.1111/acel.12720

**Published:** 2018-01-24

**Authors:** Marta Bueno, Judith Brands, Lauren Voltz, Kaitlin Fiedler, Brenton Mays, Claudette St. Croix, John Sembrat, Rama K. Mallampalli, Mauricio Rojas, Ana L. Mora

**Affiliations:** ^1^ Vascular Medicine Institute Department of Medicine University of Pittsburgh Pittsburgh PA USA; ^2^ Division of Pulmonary Allergy and Critical Care Medicine Department of Medicine University of Pittsburgh Pittsburgh PA USA; ^3^ Center for Biologic Imaging University of Pittsburgh Pittsburgh PA USA; ^4^ The Dorothy P. and Richard P. Simmons Center for Interstitial Lung Diseases University of Pittsburgh Pittsburgh PA USA; ^5^ Veterans Affairs Pittsburgh Healthcare System Pittsburgh PA USA

**Keywords:** activating transcription factor 3, aging, ER stress, idiopathic pulmonary fibrosis, mitochondrial dysfunction, PTEN‐induced putative kinase 1

## Abstract

PINK1 (PTEN‐induced putative kinase 1) is a key regulator of mitochondrial homeostasis that is relatively depleted in aging lungs and in lung epithelial cells from patients with idiopathic pulmonary fibrosis (IPF), a disease linked with aging. Impaired PINK1 expression and accumulation of damaged mitochondria in lung epithelial cells from fibrotic lungs were associated with the presence of ER stress. Here, we show that ATF3 (activating transcription factor 3), a member of the integrated stress response (ISR), negatively regulates transcription of the *PINK1* gene. An ATF3 binding site within the human *PINK1* promoter is located in the first 150 bp upstream of the transcription start site. Induction of ER stress or overexpression of *ATF3* inhibited the activity of the *PINK1* promoter. Importantly, overexpression of *ATF3* causes accumulation of depolarized mitochondria, increased production of mitochondrial ROS, and loss of cell viability. Furthermore, conditional deletion of ATF3 in type II lung epithelial cells protects mice from bleomycin‐induced lung fibrosis. Finally, we observed that ATF3 expression increases in the lung with age and, specially, in lung epithelial cells from IPF lungs. These data provide a unique link between ATF3 and PINK1 expression suggesting that persistent stress, driven by ATF3, can dysregulate mitochondrial homeostasis by repression of PINK1 mRNA synthesis.

## 1 INTRODUCTION

Idiopathic pulmonary fibrosis (IPF) is a fatal chronic lung disease characterized by progressive scarring of the lung (Raghu et al., 2011). Age dramatically increases IPF incidence and prevalence, supporting age as a well‐accepted risk factor (Raghu, Weycker, Edelsberg, Bradford & Oster, 2006). While the familial forms of IPF are primarily linked to genetic mutations, sporadic IPF cases may or may not share the same origin (Armanios et al., 2007; Lawson et al., 2004). Regardless, the mechanisms involved in aging susceptibility to IPF are still not clearly delineated (Mora, Bueno & Rojas, 2017; Selman & Pardo, 2014). One of the most well‐accepted theories in IPF pathogenesis is the vulnerability to injury of type II alveolar epithelial cells (AECIIs) which is associated with the presence of endoplasmic reticulum (ER) stress markers (Noble, Barkauskas & Jiang, 2012) and markers of accelerated epithelial cell senescence (Minagawa et al., 2011).

Recently, we have discovered that aging lungs exposed to ER stress are highly susceptible to developing mitochondrial dysfunction, similar to IPF lungs (Bueno et al., 2015; Torres‐Gonzalez et al., 2012). Hyperplasic AECIIs in IPF lungs show accumulation of swollen and dysfunctional mitochondria linked to significantly reduced levels of PINK1 (PTEN‐induced putative kinase 1). In mouse lung samples, PINK1 mRNA levels were significantly diminished with aging and exposure to the ER stressor, tunicamycin (TM) (Bueno et al., 2015). PINK1‐deficient mice showed higher frequency of enlarged swollen mitochondria associated with remodeling in the lung and high levels of the pro‐fibrotic factor TGF‐β, with a concomitant higher susceptibility to injury and fibrosis (Bueno et al., 2015). However, the mechanistic basis for the ability of ER stress to downregulate PINK1 expression is unknown.

Protein folding abnormalities induce ER stress and trigger the adaptive unfolded protein response (UPR) to halt protein synthesis to facilitate proper protein folding by increasing chaperone production. Mammalian cells present three distinct UPR signaling pathways mediated by the inositol‐requiring protein 1 (IRE1), the pancreatic ER kinase (PERK), and the activating transcription factor 6 (ATF6) acting as ER proteostasis sensors. When misfolded proteins accumulate, activated PERK phosphorylates eiF2α, attenuating global translation. Phosphorylation of eIF2α also induces preferential translation of the activating transcription factor 4 (ATF4) and subsequently activating transcription factor 3 (ATF3), both integral members of the integrated stress response (ISR) (Jiang et al., 2004).

Activating transcription factor 3 is a member of the ATF/CREB (cAMP‐responsive element‐binding protein) family of transcription factors which share a basic leucine zipper DNA binding motif and a binding consensus sequence TGACGTCA (Hai, Wolford & Chang, 2010). Transient transfection and in vitro transcription assays indicate that ATF3 represses transcription as a homodimer. Conversely, ATF3 can activate transcription when co‐expressed with its heterodimeric partners from the AP‐1 family such as c‐Jun and Chop10 (Chen, Wolfgang & Hai, 1996). ATF3 has a critical role in acute stress responses; however, its sustained activation can be detrimental (Hai, Wolfgang, Marsee, Allen & Sivaprasad, 1999)**.** This study shows a new ER‐mitochondria functional relationship, linking ATF3 to PINK1 expression. Our data suggest that ER stress, via ATF3, regulates mitochondrial homeostasis by repression of PINK1 gene transcription.

## 2 RESULTS

### 2.1 ER stress decreases PINK1 expression

**Figure 1:**
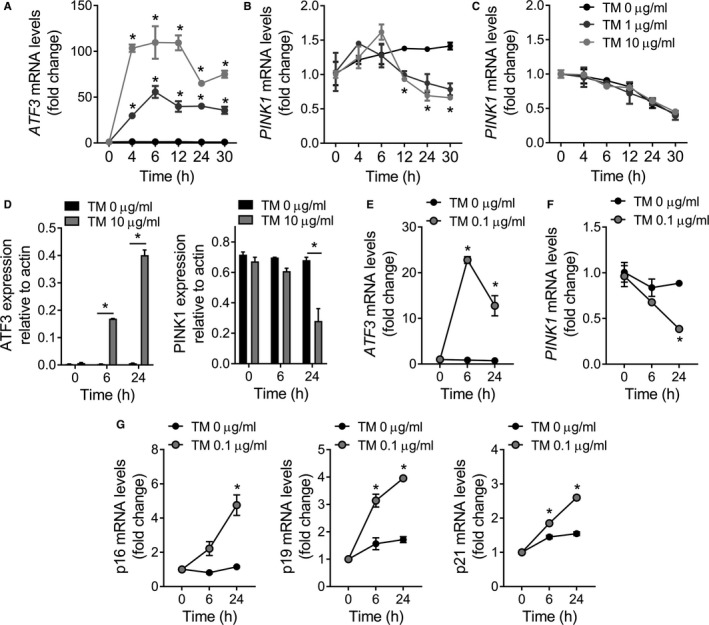
ER stress‐mediated transcriptional repression of PINK1. A549 cells show upregulation of ATF3 mRNA levels (a) after tunicamycin (TM) treatment. (b) PINK1 mRNA transcript levels are lower after TM treatment. (c) qRT‐PCR assay for PINK1 transcript stability after inhibition of transcription activity by actinomycin D does not display any differences. (d) Immunoblot analysis (see Figure S1B) of ATF3 and PINK1 protein levels at different time points after TM treatment confirmed upregulation of ATF3 and decreased PINK1. Primary human AECs exposed to low concentrations of TM show upregulation of ATF3 mRNA levels (e) and reduction in PINK1 transcript (f), concomitantly with upregulation of senescence markers (g). Data represent mean ± SEM of four (a–c) and three (d–g) independent experiments. *p < .01, two‐way ANOVA with multiple comparison test

Previously, we have shown that in vivo treatment with the ER stressor tunicamycin (TM) reduces transcript levels of PINK1 in lungs of wild‐type mice (Bueno et al., 2015). To analyze the role of ER stress in *PINK1* transcription, we treated A549 cells with tunicamycin. TM treatment induced upregulation of genes involved in the unfolded protein response (UPR) such as the ER chaperone immunoglobulin‐binding protein (BiP/Grp78, 15‐fold to 20‐fold induction), transcription factors XBP1 (fourfold to sixfold induction), CCAAT‐enhancer‐binding protein homologous protein (CHOP, 40‐fold to 80‐fold induction) (Figure S1A), and ATF3 (50‐ to 100‐fold induction) (Figure 1a). In sharp contrast, transcript levels of *PINK1* measured by qRT‐PCR were significantly reduced in A549 cells exposed to increased concentrations of tunicamycin (Figure 1b). Differences in PINK1 mRNA levels between control and TM‐treated cells were eliminated in the presence of actinomycin D (2 μg/ml), an inhibitor of transcription (Figure 1c), suggesting that ER stress mediates PINK1 transcriptional repression. These changes in relative abundance of ATF3 and PINK1 can be found at the protein level (Figure 1d, Figure S1B) and not only in A549 but also in primary human pulmonary alveolar epithelial cells (AECs). AECs exposed to a low dose of TM upregulate ER stress markers (Figure S1C). They also recapitulate the upregulation of transcript levels of *ATF3* (Figure 1e) and reduction in *PINK1* (Figure 1f). Finally, cell stress can induce premature senescence (Pascal et al., 2005; Toussaint et al., 2002), accordingly, TM‐treated AECs show increased mRNA levels of senescence markers p16, p19, and p21 (Figure 1g). Taken together, these data indicate that tunicamycin triggers UPRs in A549 and AECs and that ER stress mediates transcriptional repression of *PINK1* in epithelial cells.

### 2.2 ATF3 represses the regulator of mitochondrial homeostasis PINK1

**Figure 2:**
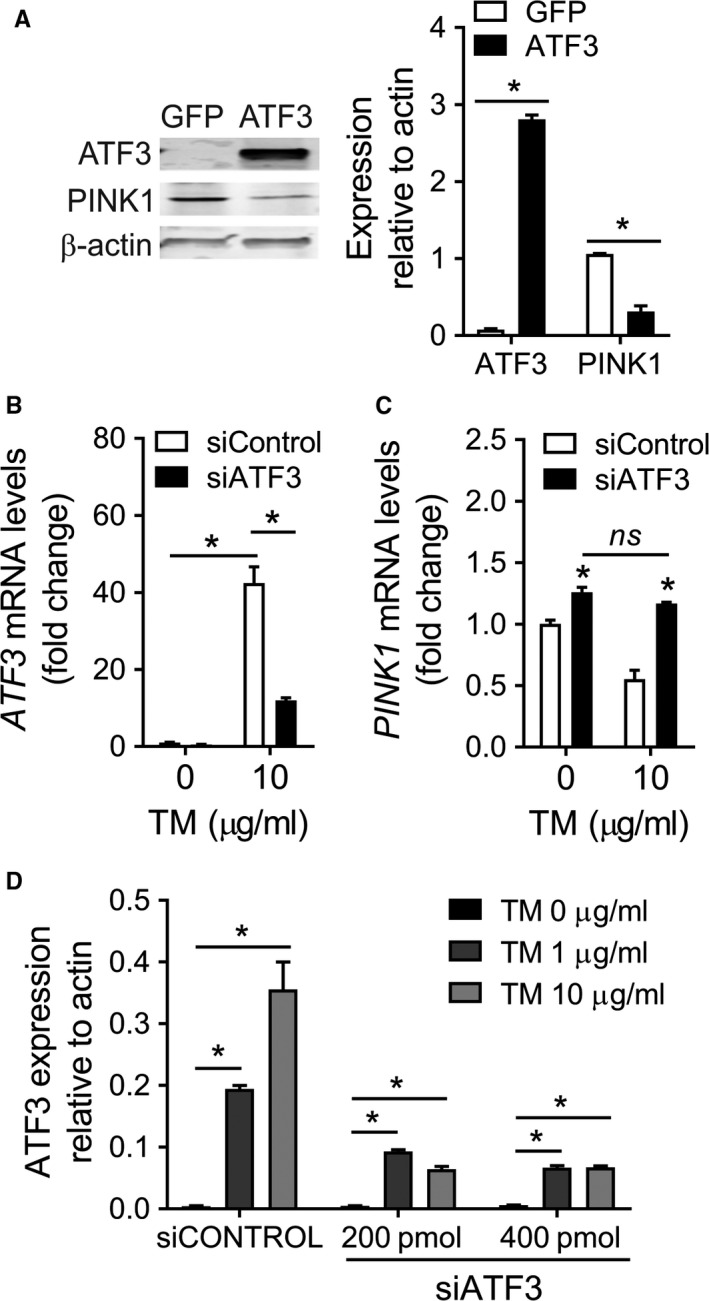
Inactivation of ATF3 potentiates PINK1 transcription. (a) Representative immunoblot analysis of ATF3 and PINK1 in total cell lysates of A549 cells, transfected with GFP (transfection control) or *ATF3*. Cells overexpressing *ATF3* for 48 hr show lower levels of *PINK1* in whole cell lysates. A549 cells transfected with siRNA scramble control or *ATF3* siRNA for a total of 48 hr and exposed to tunicamycin the last 24 hr (b–d). Less *ATF3* mRNA after 24 hr TM treatment (b) and a recovery of the basal *PINK1* transcript levels (c) were measured in knockdown ATF3 cells. (d) At 48 hr, protein levels of ATF3 also reflect these changes after TM treatment in the presence or absence of *ATF3* silencing (see Figure S2F). Data represent mean ± SEM of four (a–c) and three (d) independent experiments. *p < .01, two‐way ANOVA with multiple comparison test

Activating transcription factor 3 is a member of the ATF/CREB family of transcription factors induced by the ER stress PERK‐ATF4 pathway and, as a homodimer, can act as a transcriptional repressor (Hai & Hartman, 2001). We examined levels of PINK1 expression in A549 cells after *ATF3* overexpression. Enhanced expression of ATF3 was confirmed by immunoblotting, alongside reduction of PINK1 protein levels (Figure 2a). ATF3‐driven PINK1 reduction in vitro also drives upregulation of ER stress and fibrotic markers (Figure S2A–D) as previously shown for the PINK1‐deficient AECIIs (Bueno et al., 2015). Also, it is complemented with an increase in the senescence marker p21 (Figure S2E). To analyze whether ATF3 was required for ER stress‐mediated repression of *PINK1* transcription, A549 cells were ATF3‐depleted and transcript levels of PINK1 were measured by qRT‐PCR. Cells transfected with siATF3 showed reduced ATF3 mRNA expression before and after tunicamycin exposure (Figure 2b). Cells exposed to TM have significantly reduced PINK1 expression. Enhanced PINK1 transcript levels were observed in cells treated with siATF3 despite TM treatment (Figure 2c). Finally, siATF3 was able to reduce ATF3 protein upregulation after 24 hr TM treatment (Figure 2d, Figure S2F). These results suggest that ATF3 is required for transcriptional repression of PINK1 after ER stress induction.

### 2.3 ATF3 binds to the PINK1 promoter: characterization of the ATF3 binding sites within the PINK1 promoter

**Figure 3:**
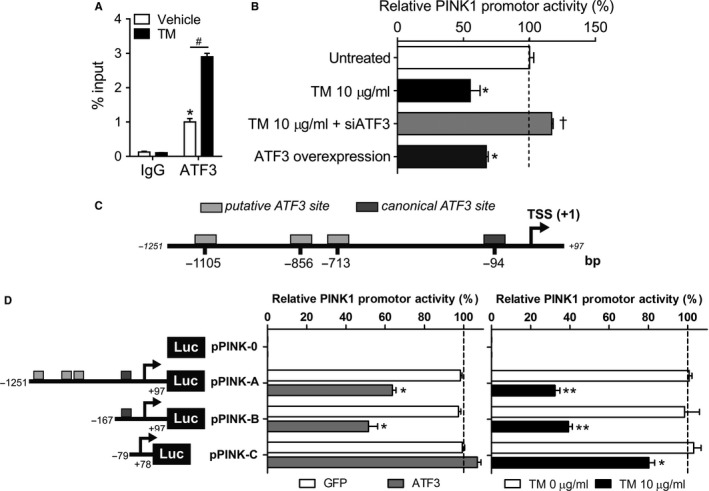
ATF3 binds to the PINK1 promoter to repress gene transcription. (a) A549 cells were treated with a low concentrations of DMSO (vehicle) or TM (1 μg/ml) for 5 hr. Chromatin immunoprecipitation (ChIP) assays on the PINK1 promoter were performed using antibodies against ATF3 and an IgG isotype control. Data represent mean ± SEM. *p < .01, two‐way ANOVA with multiple comparison test. (Negative locus, see Figure S3). (b) A549 cells transfected with a human PINK1 luciferase promoter reporter construct were treated with 10 μg/ml TM in the absence or presence of si*ATF3*, and cotransfected with an *ATF3* overexpressing plasmid. Luciferase and SEAP (secreted alkaline phosphatase) activities were measured after 24‐hr stimulation. (c) Schematic diagram of the 1.2 kb PINK1 promoter region in the 5′ flanking region upstream of the transcriptional starting site (TSS). (d) Deletion constructs of the 1.2 kb cloned PINK1 promoter luciferase reporter plasmid (pPINK‐A). Arrows represent the direction of transcription and the numbers detail the endpoint of each construct. The deletion plasmids were transfected in A549 cells, and after 24 hr of TM (10 μg/ml) treatment or overexpression of *ATF3* plasmid (or GFP as control), promoter activity was measured. Luciferase activities were normalized to SEAP activities. Values obtained for the untreated (or GFP cotransfected) sample of each construct represent 100%. Data are reported as mean ± SEM of four independent experiments. One‐way ANOVA with multiple comparison test; *p < .05 vs. untreated, ^†^ *p* < .01 vs. TM 10 μg/ml (b). *p < .05 and **p < .01, unpaired, two‐tailed Student's *t* test vs. each corresponding untreated (or GFP cotransfected) sample (d)

Next, we evaluated interaction of endogenous ATF3 to the *PINK1* promoter in A549 cells. Chromatin immunoprecipitation (ChIP) assay performed with an anti‐ATF3 antibody showed positive binding of ATF3 to the *PINK1* promoter. ChIP assays performed with a pre‐immune IgG detected no such enrichment of ATF3. Signals obtained in the input sample were used to normalize the data. ATF3 binding on the *PINK1* promoter was positive in cells treated with vehicle control and significantly increased following TM (1 μg/ml) stimulation (Figure 3a, Figure S3A). These data show that ATF3 binds to the *PINK1* promoter under ER stress conditions.

The human *PINK1* promoter activity was monitored in A549 cells using a Gaussia luciferase reporter driven by a sequence of 1,251 bases located upstream of the transcription starting site (TSS) of PINK1. Reduced luciferase activity was observed after 24 hr exposure to TM when compared with its basal activity (Figure 3b). The internal control reporter (SEAP, secreted alkaline phosphatase under a generic CMV promoter) activity was not significantly altered after TM treatment (Figure S3B). PINK1 promoter activity was fully restored when the cells were depleted of ATF3 before TM exposure (Figure 3b). On the other hand, overexpression of *ATF3* showed decreased activity of the PINK1‐driven luciferase reporter (Figure 3b) compared with nontransfected or GFP‐transfected cells (Figure S3C–D). These results indicate that the PINK1 promoter is repressed by ER stress agonists mediated, in part, through ATF3.

Sequence analyses and a computer‐based transcription factor binding site search (TRANSFAC, http://www.genexplain.com) of the 1.2‐kb promoter region of the human PINK1 gene reveal the presence of one canonical ATF3‐responsive element site (plus three other putative ones) within the 5′ flanking region upstream of the transcription start site (TSS) of the PINK1 gene (Figure 3c). The four possible ATF3 elements in the 1.2‐kb PINK1 promoter region are located at −1,105, −856, −713, and −94 base pairs from the TSS, respectively. The sequences are homologous to the ATF3 consensus sequence TGACGTCA. A series of deletion mutants within the 1.2‐kb fragment of the promoter reporter plasmid (*pPINK‐A*) were generated to identify the core promoter required for PINK1 gene expression and the activity of the ATF3 sites. Cellular expression of a *pPINK‐0* plasmid that harbored the reporter gene but no promoter, as expected, exhibited no activity (Figure 3d). Luciferase activity assays in cells transfected with the full‐length promoter (*pPINK‐A*) or with a construct lacking the −1,251 to −167‐bp region in the PINK1 promoter (*pPINK‐B*) displayed similar lower luciferase activities after TM (10 μg/ml) treatment or with ectopic expression of *ATF3*. The *pPINK1‐B* construct contains the 3′ *cis*‐acting site for ATF3 in the PINK1 promoter, suggesting that this site is sufficient to induce ATF3 repression of PINK1 gene transcriptional activity **(**Figure 3d). No inhibition of activity by *ATF3* was observed in cells expressing the −78 to +28 PINK1 promoter sequence (*pPINK‐C*) in line with previous studies (Duan et al., 2014) that identified this region as the minimal PINK1 promoter (Figure 3d). The slight TM‐mediated inhibition of this minimal PINK1 promoter sequence is probably due to the activation of other ER stress pathways leading to global cell transcription arrest (that are not activated when only overexpressing ATF3). Altogether, these data support that a key ATF3 binding site is located in the first 150 bp upstream PINK1 transcription start site.

### 2.4 Overexpression of ATF3 compromises mitochondria homeostasis

**Figure 4:**
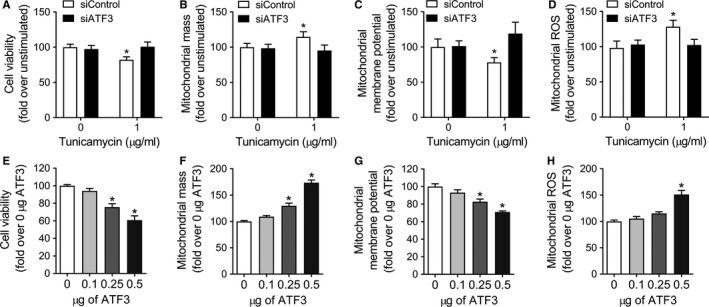
ATF3 modulates mitochondrial homeostasis and cell viability. ATF3 was silenced in A549, and experiments were performed after 24 hr of tunicamycin treatment. (a) Hoechst 33342 staining was used to assess cell viability. Cell transfected with siATF3 did not lost cell viability under TM exposure. Mitochondrial mass (b) and depolarization (c) were assessed by MitoTracker and JC‐1 staining, respectively. *ATF3* silencing was beneficial to the injured cells, showing complete recovery from the ER stress‐induced accumulation of depolarized mitochondria. (d) Mitochondrial ROS generation, by MitoSOX staining, was also rescue in the knockdown *ATF3* cells. A549 cells were transfected with different quantities of *ATF3* plasmid for 48 hr, and cell viability and mitochondrial health were measured. (e) Transfected cells showed a dose‐dependent cell death after exposure to high amounts of ectopically expressed *ATF3*. *ATF3* overexpression was detrimental to the cells, showing a dose‐dependent accumulation (f) of depolarized mitochondria (g). Mitochondrial ROS generation (h) was also elevated in the cell overexpressing *ATF3*. All measurements were taken 48 hr after transfection. Data represent mean ± SEM of 24 replicates per condition, in three independent experiments. *p < .01 vs. untreated, one‐way ANOVA with multiple comparison test

We examined whether the modulation of ATF3 expression could rescue (by silencing) or mimic (by overexpression) the accumulation of depolarized mitochondria observed with ER stress or in PINK1‐deficient cells (Bueno et al., 2015). To analyze mitochondria homeostasis under those conditions, ATF3‐silenced A549 were treated with TM for 24 hr (Figure 4a–d), and also, A549 were independently transfected with increasing amounts of *ATF3* expression plasmid (Figure 4e–h) or transfection plasmid control (Figure S4). As we previously reported, TM treatment caused decreased in cell viability and mitochondrial membrane potential, and increase in mitochondrial mass and production of mitochondrial ROS (Bueno et al., 2015). This phenotype was reversed in the absence of ATF3. A549 cells transfected with siATF3 and treated with TM do not show a reduction in cell viability neither the accumulation of depolarized, ROS‐producing mitochondria (Figure 4a–d). In addition, cell viability was reduced in the *ATF3*‐overexpressing cells at high concentration (Figure 4e). Transfected cells also showed dose‐dependent mitochondrial accumulation (Figure 4f) with significant depolarization (Figure 4g). In parallel, *ATF3* overexpression in cells exhibited higher levels of mitochondrial ROS (Figure 4h). In summary, these data corroborate that upregulation of ATF3, present in persistent ER stress, can affect mitochondrial homeostasis through PINK1‐reduced transcription. Furthermore, the in vitro phenotype previously reported (Bueno et al., 2015) can be rescued by silencing ATF3.

### 2.5 ATF3 is upregulated in fibrotic and aged mice lungs

**Figure 5:**
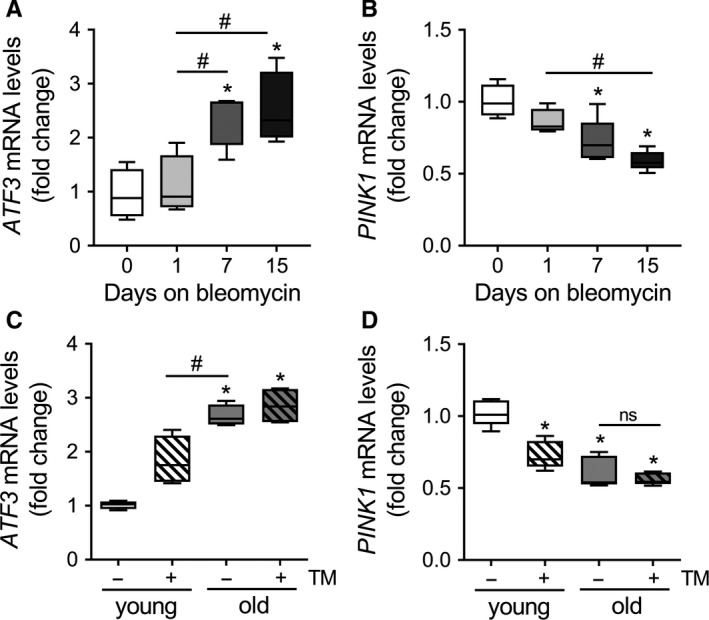
Upregulation of ATF3 in bleomycin‐induced fibrotic lung and in the lung of the aging. Young mice (3 months old) were treated with 1.5 U/kg of bleomycin intratracheally and their lungs were harvested at different time points. In total lung lysate, mRNA transcript levels of ATF3 (a) are upregulated while levels of PINK1 are downregulated (b). When comparing young (3 months old) vs. old (20 months old) mice, the old mice present elevated levels of ATF3 (c) concomitantly with lower PINK1 transcript (d). After treating young mice with tunicamycin (TM) intratracheally, their lungs show similar mRNA levels of ATF3 (c) and PINK1 (d) as the old mice. Data represent mean ± SEM of *n *=* *6. (a–c) *p < .01 vs. day 0 and ^#^ *p* < .01 as indicated, one‐way ANOVA with multiple comparison test. (c–d) *p < .01 vs. young and ^#^ *p* < .01 as indicated, two‐way ANOVA with multiple comparison test

To examine the role of ATF3 in the fibrotic lung, young mice (3 months old) were treated with 1.5 U/kg of bleomycin intratracheally and their lungs were harvested at different time points (Figure S5). These mice start upregulating fibrotic markers, such as *COL1A1* and *FN1* (Figure S5A–B) by day 7, and collagen deposition was detected at day 15 by hydroxyproline assay (Figure S5C). In total lung lysate of the bleomycin‐treated mice, as the fibrotic injury sets, mRNA transcript levels of *ATF3* (Figure 5a) are upregulated while levels of *PINK1* are downregulated (Figure 5b).

To investigate the role of ATF3 in lung aging, we compared total lung lysate from young (3 months old) and old (20 months old) mice. The old mice present elevated levels of ATF3 (Figure 5c) concomitantly with lower PINK1 transcript (Figure 5d). After treating young mice with tunicamycin (TM) intratracheally for 15 days, their lungs show similar mRNA levels of ATF3 (Figure 5c) and PINK1 (Figure 5d) as the old mice. In summary, these data suggest a role for ATF3 upregulation in the pathogenesis of lung fibrosis and in the aging lung.

### 2.6 Conditional and selective ATF3 deletions from mice AECIIs protect from bleomycin‐induced lung fibrosis

To explore the potential role of ATF3 on epithelial injury and senescence in vivo, we generated an inducible conditional type II lung epithelial cells ATF3 knockout mice (ATF3 spc‐KO) and recombination was confirmed after 2 to 4 weeks of doxycycline administration (625 mg/kg) in chow (Figure S6). Recombination was positive in the lung AECIIs (Figure S6A and C) and was not detected in other tissues, such as liver (Figure S6B).

**Figure 6:**
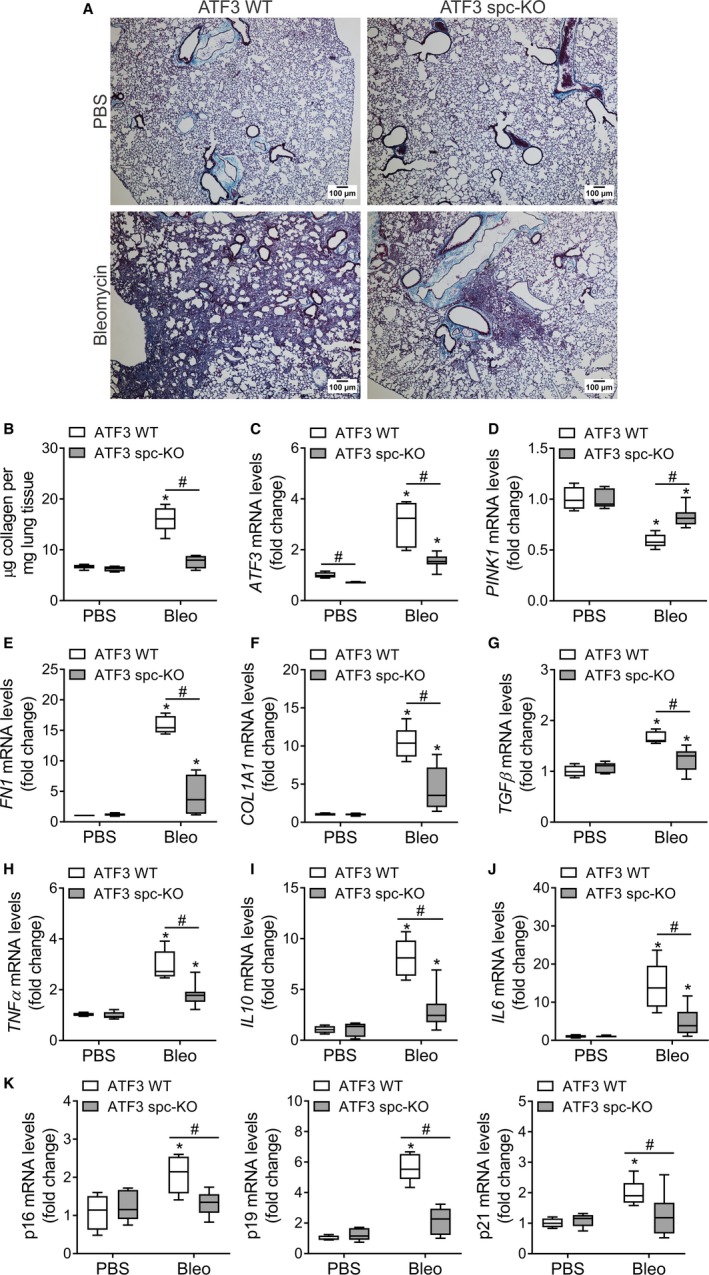
ATF3 increases susceptibility to lung fibrosis. (a) Representative Masson trichrome staining in lung sections from ATF3 WT and ATF3 spc‐KO (conditional type II lung epithelial cells ATF3 knockout mice) showing decreased collagen deposition (blue) at day 15 postbleomycin instillation. (b) Decreased collagen deposition in lungs of ATF3 spc‐KO mice after bleomycin determined by hydroxyproline levels. (c) Bleomycin‐mediated upregulation of ATF3 mRNA in the lung of the WT mice is reduced in the ATF3 spc‐KO mice, hand‐in hand, with an improvement in PINK1 transcript levels (d). Higher fibronectin (e) and collagen I (f) transcript levels in lungs of the WT mice compared to the AECII‐specific KO littermates. (g) Relative change in the levels of TGF‐β transcripts. Relative change in (h) TNF‐α, (i) IL‐10, and (j) IL‐6 mRNA levels after bleomycin treatment in ATF3 WT and ATF3 spc‐KO mice. (k) ATF3 spc‐KO mice present lower levels of senescence markers in total lung lysate. Data represent mean ± SEM of *n *=* *6–8. (b–k) *p < .01 vs. ATF3 WT PBS and ^#^ *p* < .01 as indicated, two‐way ANOVA with multiple comparison test

Intratracheal instillation of bleomycin (1.5 U/kg) was performed in ATF3 WT, ATF3 fl/fl, and ATF3 spc‐KO mice (3 months). ATF3 spc‐KO show reduced lung pathology (Figure 6a) and decreased collagen deposition at day 15 postbleomycin treatment by Masson trichrome. Total lung collagen was corroborated by hydroxyproline assay, showing more collagen in ATF3 WT and ATF3 fl/fl at day 15 postinjury (Figure 6b, Figure S8A). Even in the areas where the pathology is more severe, collagen deposition in the ATF3 spc‐KO mice is dramatically reduced when compared with ATF3 WT (Figure S7A). ATF3 spc‐KO mice are not able to upregulate ATF3 expression in the AECIIs, and the ATF3 transcript levels in total lung lysate are lower (Figure 6c, Figure S8B) and can preserve a higher expression of PINK1 even after injury (Figure 6d, Figure S8C). Additionally, expression of collagen and fibronectin was reduced in the ATF3 spc‐KO mice at day 15 postbleomycin treatment (Figure 6e–f, Figure S8D–E). Moreover, ATF3 WT and ATF3 fl/fl lung showed higher levels of the pro‐fibrotic mediators TGFβ and FGF2 (Figure 6g; Figures S7B, S8F–G).

Inflammatory responses were also more severe in the ATF3 WT and ATF3 fl/fl control mice than those in the ATF3 spc‐KO littermates, as determined by BAL cell counts (Figures S7D, S8L) and the changes in mRNA levels of the cytokines TNFα, IL6, and IL10 (Figure 6h–j, Figure S8H–J), despite IL1β not differing significantly among the groups (Figures S7C, S8K). Finally, the pro‐senescence milieu in the WT bleomycin‐treated mice lung (Figure S5E) can be related with the downregulation of PINK1 (Figure 5b). PINK1‐deficient mice show higher transcript expression of p16, p19, and p21 even at young age (Figure S9A–C). Senescence markers in the ATF3 spc‐KO lungs were strikingly decreased at day 15 after bleomycin instillation when compared with WT of fl/fl littermates (Figure 6k, Figure S8M) and can be associated with the preservation of PINK1 expression after injury (Figure 6d). Altogether, these data suggest that ATF3, via regulation of PINK1, has an important role in the AECIIs susceptibility to lung injury, senescence, and development of lung fibrosis.

### 2.7 IPF is associated with enhanced expression of ATF3

**Figure 7:**
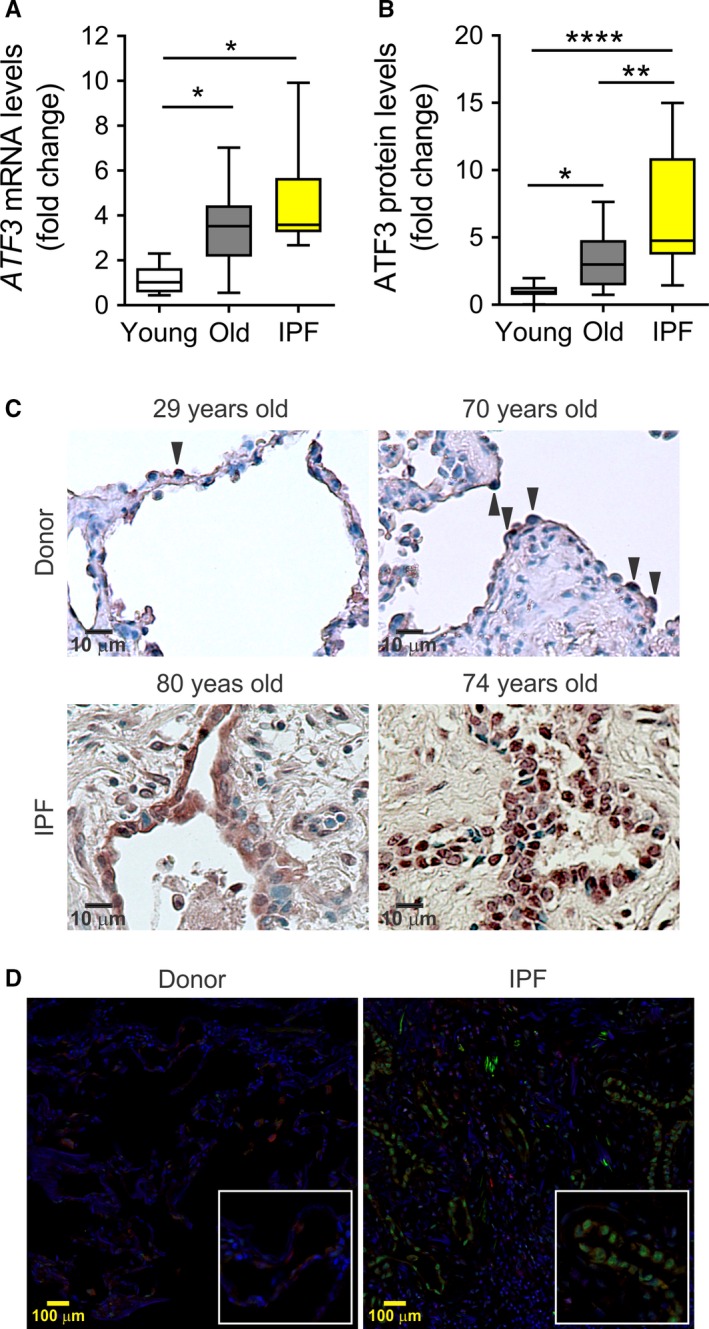
Upregulation of ATF3 with aging and in idiopathic pulmonary fibrosis (IPF) lungs. Analysis of ATF3 expression in total lung lysates of young donor (<50 years old), old donor (more than 50 years old), and IPF lungs (*n *=* *17 patients per group). Significantly increased ATF3 expression in old donor and IPF tissue was detected by mRNA (a) transcript levels and protein densitometry (b). Data represent mean ± SEM. *p < .01, **p < .01, and *^**^ *p* < .001 vs. young donor, one‐way ANOVA with multiple comparison test. (c) Representative images (*n *=* *3) of immunohistochemistry analyses from young and old donors and IPF lungs using ATF3 antibody. Arrows denote epithelial cells showing nuclear staining. (d) Representative immunofluorescence using anti‐ABCA3 (type II epithelial cell marker; red) and anti‐ATF3 (green) antibodies plus DAPI to stain nuclei blue, showing high ATF3 expression in the nuclei of hyperplasic AECIIs from honeycombs in IPF lung

Markers of ER stress have been shown to be highly expressed in AECII of the IPF lung. ATF3 is induced by ER stress, but no previous studies have shown whether ATF3 is expressed in lungs of IPF patients. We analyzed ATF3 expression in whole lung lysates of young (<50 years), old (>50 years), and IPF patients (detailed in Table S1). Levels of *ATF3* transcript and immunoblot analyses (Figure S10A) in total lung lysates showed that ATF3 is highly expressed in IPF lungs in comparison with young and old age‐matched control donor lungs (Figure 7a–b). Using immunohistochemistry studies, we confirmed the presence of nuclear ATF3 in lung epithelial cells in aging normal lungs (Figure 7c). In addition, strong nuclear staining was found in AECII lining honeycomb areas in IPF lungs (Figure 7c). We confirmed that in fact, in the honeycombing areas of the IPF lung, the ATF3 overexpressing lung cells are alveolar type II epithelial cells by colocalization of ATF3‐ and ABCA3‐positive staining (Figure 7d, Figure S10B). These data suggest that high expression of ATF3 might be relevant in aging cell perturbations in lung epithelial cells and in the pathogenesis of lung fibrosis.

## 3 DISCUSSION

Recently, we unveiled a key role for PINK1 in the maintenance of mitochondrial quality control in AECII and in activation of pulmonary pro‐fibrotic responses (Bueno et al., 2015). Levels of this homeostatic protein diminish with age and are greatly reduced in IPF lungs. Here, we demonstrated that ER stress transcriptionally inhibits expression of PINK1 in lung epithelial cells, recapitulating the finding in IPF and aging lungs. ER stress triggers the UPR with concomitant induction of ATF3, a known transcriptional repressor. ATF3 binds to the PINK1 promoter, serving as a putative PINK1 transcriptional repressor. In contrast, knockdown of *ATF3* in lung epithelial cells under ER stress partially restores PINK1 expression. In addition, overexpression of *ATF3* compromises mitochondrial homeostasis leading to accumulation of unhealthy, depolarized mitochondria that are characterized by higher mitochondrial ROS generation.

Activating transcription factor 3 expression increases with age and fibrosis, suggesting that this factor may play a crucial role in age‐related increased susceptibility to mitochondrial dysfunction and lung fibrosis. Conditional type II lung epithelial cells ATF3 knockout mice are protected from severe lung fibrosis induced by bleomycin showing a better outcome measured by pro‐fibrotic, pro‐inflammatory, and senescence markers while preserving PINK1 expression in the lung. These data strongly suggest that ATF3, via regulation of PINK1, has a key role in the AECIIs susceptibility to lung injury, senescence, and development of lung fibrosis.

The decline of proteostasis with aging leads to an increase in nonfunctional proteins, accumulation of cytotoxic proteins, and/or aggregation of misfolded proteins with subsequent increased vulnerability to cell stress. It is recognized that the ER and mitochondria cooperate to balance pro‐survival and pro‐death pathways. ER and mitochondria interact physiologically and functionally that mediate key cell responses functions such as calcium signaling, inflammation, lipid metabolism, biogenesis, apoptosis, and autophagy (Herrera‐Cruz & Simmen, 2017). This interaction is a component of the integrated stress response (ISR), a system that coordinates communication between the nucleus and mitochondria during mitochondrial homeostasis and stress (Quiros, Mottis & Auwerx, 2016). A key regulator of the ISR is the activation of the α‐subunit of eukaryotic translation initiation factor 2 (eIF2α) that inhibits protein synthesis and simultaneously facilitates the expression of specific stress response genes such as ATF4 and ATF3. Expression of ATF3 triggers rapid induction of a large number of target genes to restore proper cellular function (Hai et al., 1999); however, the ISR has a hormetic feature and depending on the severity of stress this can lead to pro‐survival or pro‐apoptotic outcomes, thus compromising mitochondrial homeostasis. Our data suggest that ATF3 is a key component of the ISR in the lung epithelial cells that modulates mitochondrial stress through PINK1.

Activating transcription factor 3 expression increases with a wide spectrum of cellular stresses including ER stress, DNA damage, nutrition deprivation, hypoxia, ROS, TLR activation, TGF‐β, IFN, HDL, and wounding. Thus, ATF3 is a critical adaptive response gene in different cells and organs. For instance, ATF3 is required to adjust glucose metabolism in mice exposed to high‐fat diet (Zmuda et al., 2010). ATF3 also promotes cell stress‐induced senescence by regulation of the Id1 transcription factor that control p16 expression (Chambers, Leoni, Kaminski, Laurent & Heller, 2003; Hara et al., 1994). In addition, ATF3 is cardioprotective during hypertrophy induced by increased pressure loads (Zhou et al., 2011). However, after unresolved stress, sustained overexpression of ATF3 can be detrimental and contribute to disease, including fibrosis. Fibrotic diseases are believed to be associated with a failure to adequately contain or eliminate inciting injury factors. One sequel is exaggerated activation of repair responses and abnormal accumulation of extracellular matrix components that leads to organ dysfunction and failure. Our data show that ATF3 expression is increased in fibrotic human lungs. Interestingly, it has been shown that ATF3 overexpression promotes spontaneous hypertrophy of the heart and cardiac fibrosis (Koren, Elhanani, Kehat, Hai & Aronheim, 2013). On the other hand, ATF3‐deficient mice are protected from cardiac hypertrophy and bleomycin‐induced skin fibrosis (Koren et al., 2013; Mallano et al., 2016).

PTEN‐induced putative kinase 1 initially was identified as a regulator of mitochondrial quality control pathway implicated with Parkin in the mitophagy process. However, the identification of novel targets for PINK1's serine/threonine kinase activity indicate that PINK1 has a more extensive role in mitochondrial homeostasis, including mitochondrial fusion/fission dynamics, mitochondrial proteostasis, mitochondrial trafficking, modulation of mitochondrial respiration, calcium mobilization, and apoptosis. Changes in PINK1 affect mitochondrial quality control according to age, cell type, and severity of stress, indicating that energy demand and adaptation capacity to stress modulate the vulnerability to PINK1 deficiency. FOXO3A, NF‐κB, and Nrf2 have been identified as positive regulators of transcription of the PINK1 gene in the mouse and human promoters, respectively (Duan et al., 2014; Mei et al., 2009; Murata et al., 2015). Interestingly, others and we have shown that PINK1 expression decreases with age (Bueno et al., 2015; Sosulski et al., 2015). In parallel, we have found ATF3 expression is higher in aging human lungs. Although further studies are necessary, it will be of interest to determine whether aging‐associated mitochondrial dysfunction might be linked to age‐related increase in ATF3 expression and repression of PINK1.

PTEN‐induced putative kinase 1 expression has been found also to be induced by the canonical PTEN and its isoform PTENα (Liang et al., 2014). Fibroblasts and epithelial cells from IPF lungs have been found that express low levels of PTEN with persistent Akt activation (Miyoshi et al., 2013; Xia et al., 2008, 2010). PINK1 expression is also induced by TGFβ in lung epithelial cells, and immunofluorescence studies in the IPF lung have shown positive staining for PINK1 protein potentially associated with the accumulation of damaged mitochondria (Patel et al., 2015). Nevertheless, in vivo deficiency of PINK1 results in increased susceptibility to fibrosis and TGF‐β1‐induced cell death (Patel et al., 2015).

In conclusion, in addition to ER stress, ATF3 is a hub of adaptive stress responses but also in age‐related cell stress (Kim, Park, Rhee & Pyo, 2015). It is a versatile transcription factor in which activity and outcome are deeply dependent on the biological context of the stress, severity, and persistence. Acute upregulation of ATF3 is in most cases beneficial; however, sustained expression of ATF3 due to unresolved stress is usually linked with pathological states. The data presented here link ATF3 to PINK1 expression which in turn suggests that ER stress, via ATF3, regulates mitochondrial homeostasis by repression of PINK1 gene transcription. This connection may be critical in devising strategies designed to augment PINK1 expression or inhibit ATF3 levels to lessen severity of pulmonary fibrosis, especially in the context of the aging lung.

## 4 EXPERIMENTAL PROCEDURES

### 4.1 Cell culture, maintenance, and treatments

Human lung adenocarcinoma cells (cell line A549, ATCC CCL‐185) were cultured in DMEM (Gibco) with 10% FBS (Gibco) and 50 U/ml penicillin with 50 μg/ml streptomycin (Gibco) in 5% CO2 at 37°C. A549 cells were treated with TM (1 or 10 μg/ml; Sigma‐Aldrich) alone for 24 hr, using DMSO (0.02%; Sigma‐Aldrich) as vehicle control (TM 0 μg/ml). For ATF3, GFP, or V5‐tag overexpression, cells were transfected with pRK‐ATF3 (Addgene), pGFP‐V‐RS (Origene), or pLenti6/V5‐DEST (Thermo Fisher), respectively. To knock down ATF3 expression, cells were transfected with siRNA for ATF3 (sc‐29757, Santa Cruz) or scramble siRNA controls (Select Negative Control No. 2 siRNA, Thermo Fisher 4390847). Treatments were performed beginning 24 hr after transfection and for an additional 24 hr. To analyze the PINK1 mRNA degradation, A549 cells were treated with 2 μg/ml of actinomycin D (Sigma)—for the duration of the experiment—in the presence or absence of the tunicamycin (1 or 10 μg/ml).

Primary human pulmonary alveolar epithelial cells (AECIIs, ScienCell #3200, cryopreserved at passage 0) were seeded in a poly‐L‐lysine coated plate (ScienCell #0403). Cells grew overnight in complete alveolar epithelial cell medium (ScienCell #3201) and then treated with tunicamycin (0.1 μg/ml; Sigma‐Aldrich) or vehicle control (DMSO 0.002%, Sigma‐Aldrich) for 0, 6, or 24 hr.

### 4.2 Animals and animal treatment

All animal protocols were approved by the IACUC of the University of Pittsburgh and adhered to NIH guidelines for the use of experimental animals. Mice with ATF3 exon 2 coding region flanked with LoxP recombination sites (ATF3 fl/fl) and mice carrying a doxycycline‐inducible Cre recombinase under the control of a regulatory sequence of the SPC gene (C57BL/6J‐Tg[tetO‐cre]/Tg[SFTPC‐rtTA]) were bred to generate homozygous‐inducible conditional type II lung epithelial cells ATF3 knockout mice (ATF3/tetO‐cre/SFTC‐rtTA). The ATF3 fl/fl mice were obtained from Dr. Tsonwin Hai (Department of Molecular and Cellular Biochemistry, The Ohio State University) (Hartman et al., 2004; Wolford et al., 2013). All mice used in this study were genotyped by PCR to detect ATF3 fl/fl, tetO‐cre, and SFTPC‐rtTA sequences (details in Supporting Information). To promote recombination and generate a homozygous conditional type II lung epithelial cells ATF3 knockout mice (ATF3 spc‐KO), mice were fed doxycycline chow (625 mg/kg, Envigo) for 2 weeks before injury induction.

For the tunicamycin‐induced ER stress experiments, young (3 months) and old (20 months) C57BL/6 (Jackson Laboratories and National Institute of Aging) were treated with tunicamycin or vehicle only. TM was dissolved in DMSO (5 mg/ml) and then diluted 1:1,000 in PBS for intratracheal injection. Mice were sacrificed for organ harvest 15 days after the procedure. Control mice received an intratracheal dose of 1:1,000 DMSO dilution in sterile PBS.

For the bleomycin‐induced fibrosis experiments, a dose of 1.5 U/kg of bleomycin dissolved in sterile PBS (administered intratracheally) was used in 3‐month‐old ATF3 WT, ATF3 fl/fl, and ATF3 spc‐KO mice. Mice were sacrificed for organ harvest at day 0, 1, 7, and 15 (ATF3 WT) or at 15 postinstillation (ATF3 fl/fl and ATF3 spc‐KO). Control mice received an intratracheal dose of sterile PBS. Mice were fed doxycycline chow through the duration of the bleomycin experiment. Unless otherwise specified, six to eight mice per group were analyzed in these experiments.

### 4.3 Quantitative PCR (qPCR)

RNA was extracted using RNeasy kit (Qiagen) according to manufacturer's protocol. Quantitative real‐time reverse transcription‐PCR (qRT‐PCR) was then performed as previously described (Bueno et al., 2015). Data analysis was based on the 2^−ΔCT^ method using RNA18S as normalization control. The primers used for the real‐time PCR are listed in Table S2. PCR was conducted in a QuantStudio5 real‐time PCR system (Thermo Fisher) and analyzed using QuantStudio Design & Analysis software v1.3.1 (Thermo Fisher).

### 4.4 Immunoblot analysis

Protein extraction reagents, MPER or TPER (Thermo Fisher), were used to prepare the protein extracts. Around 20 μg of total lysate was used for electrophoresis in a polyacrylamide gel. Proteins were transferred to a PVDF membrane and blocked for 1 hr with Odyssey PBS blocking buffer (Licor). Primary antibodies for ATF3 (sc‐188, Santa Cruz) and PINK1 (BC100‐494, Novus) were incubated overnight. Finally, secondary antibodies (Licor) and Odyssey scanner (Licor) were used. After primary detection, membranes were stripped and re‐probed for β‐actin (sc‐47778, Santa Cruz) as loading control. Quantification was performed using ImageJ software (NIH).

### 4.5 Chromatin immunoprecipitation assay

Chromatin immunoprecipitation (ChIP) assay was performed as described in the Supplemental information. Real‐time PCR was performed using SYBR Green‐based detection (Promega) using equal amounts of ChIP and diluted input DNAs. ChIP antibodies included anti‐ATF3 (sc‐188 X, Santa Cruz) and an IgG control antibody (10500C, Pierce).

### 4.6 Human PINK1 promotor luciferase reporter plasmids and assay

The PINK1 promotor luciferase reporter plasmid (*pPINK1‐A*) was custom‐made using the pEZX‐PG04 vector (GeneCopoeia, HPRM22074‐PG04). The total promoter length clones was 1,353, 1,259 bp upstream of the transcription starting site (TSS), and 93 pb downstream of the TSS. The different deletions of *pPINK‐A* were constructed as follows. The 1,353 bp of the PINK1 promoter was eliminated by PCR to produce the pPINK‐0 plasmid. To obtain the promoter deletions *pPINK‐B* (**−**167 to +97 bp) and *pPINK‐C* (**−**79 to +78 bp), we used gBlocks gene fragment from Integrated DNA Technologies (Coralville, IA). Those fragments were flanked with HindIII and EcoRI restrictions sites to be inserted on the same PG04 plasmid background.

A549 cells were transiently transfected with each of the luciferase reporter plasmid and cotransfected with pRK‐ATF3 (ATF3 overexpression) or pGFP‐V‐RS (GFP overexpression) or treated with tunicamycin (1 or 10 μg/ml). After 48 hours of transfection and 24 hr post‐treatment, the cell culture medium was collected. Secrete‐Pair Dual Luminescence Assay Kits (GeneCopoeia) were used to detect secreted alkaline phosphatase (SEAP) and secreted Gaussia luciferase (GLuc) activities using the manufacturer's protocol. The SEAP signal was used as a transfection efficiency internal control. Luminescence intensities were detected using a Synergy Microplate Reader (BioTek). The normalized GLuc activity (GLuc/SEAP ratio) of all samples was compared.

### 4.7 Mitochondrial functional assays

For the silencing experiments, a total of 10^4^ A549 cells were seeded on 96‐well plates, cultured overnight in DMEM (high glucose; Gibco) with 5% FBS (Gibco), and then transfected with siRNA scramble control of siATF3. Media were replaced with completed DMEM (DMEM with 10% FBS, 50 U/ml penicillin with 50 μg/ml streptomycin) with or without 10 μg/ml of tunicamycin 24 hr after transfection, and cells were grown for another 24 hr. For overexpression experiments, a total of 10^4^ A549 cells were seeded on 96‐well plates, cultured overnight in DMEM (high glucose; Gibco) with 5% FBS (Gibco), and then transfected with different amounts of *pRK‐ATF3* (ATF3 overexpression) or *pLenti6/V5‐DEST* (V5‐tag overexpression). Media were replaced with complete DMEM 24 hr after transfection, and cells were grown for another 24 hr. All measurements were acquired 48 hr after the initial transfection by bottom read in a Synergy Microplate Reader (BioTek). Hoechst 33342 (Thermo Fisher) was used to calculate the viability. JC1 Dye (T3168, Thermo Fisher) was used to assess mitochondrial membrane potential. Mitochondrial mass and mitochondrial reactive oxygen species were calculated using MitoTracker Deep Red (M22426, Thermo Fisher) and MitoSOX (M36008, Thermo Fisher), respectively. Details are provided in the Supporting Information.

### 4.8 Hydroxyproline assay

Dried‐pulverized frozen lung tissue samples (~5 mg per sample) were resuspended in 10 volume of deionized water and 10 volume of 12N HCl, and then hydrolyzed at 110°C overnight. Assay reactions were performed following the manufacturer's protocol (Sigma). A standard curve was generated using trans‐4‐hydroxy‐L‐proline (Sigma). Results were expressed as micrograms of collagen per milligram of lung tissue.

### 4.9 Immunohistochemistry and immunofluorescence staining

After sacrifice, mice lungs were perfused with 2% paraformaldehyde followed by paraffin embedding. Paraffin sections were stained with Masson trichrome to determine collagen deposition. Collected human lungs samples were perfused with 2% paraformaldehyde, followed by saturation in 30% sucrose for frozen sections or paraffin embedding. Antibodies against ATF3 (HPA001562, Sigma) were used to perform the immunohistochemistry studies in paraformaldehyde fixed paraffin embedded lung tissue. Immunofluorescence analyses were performed in paraformaldehyde‐fixed sucrose‐saturated frozen sections of lung tissue using antibodies against ATF3 (HPA001562, Sigma) and ABCA3 (WMAB‐ABCA‐17, Seven Hills).

### 4.10 Statistical analysis

Statistical analysis was performed using Prism 7 (GraphPad). Differences between groups were calculated by two‐tailed Student's *t* test or by one‐ or two‐way ANOVA followed by post hoc tests. Results are presented as mean ± SEM. *p*‐Values less than .05 are considered significant.

### 4.11 Study approval for human samples

Human lung tissue was collected from excess pathologic tissue after lung transplantation and organ donation, under University of Pittsburgh IRB‐ and CORID‐approved protocols (970946, PRO14010265, and CORID No. 300).

## CONFLICT OF INTEREST

None declared.

## AUTHOR CONTRIBUTIONS

All authors participated in the revision of the manuscript. MB, RKM, and ALM performed study conception and design. MB, JB, LV, KF, BM, CSC, and JS carried out acquisition of data. MB, RKM, MR, and ALM carried out analysis and interpretation of data. MB, MR, and ALM drafted the manuscript.
