# Supplementary material for: ATF3 represses PINK1 gene transcription in lung epithelial cells to control mitochondrial homeostasis
Source: Aging Cell. 2018 Jan 24;17(2):e12720. doi: 10.1111/acel.12720 (PMC5847866; doi:10.1111/acel.12720)
Supplement: Supplementary file 1 [file ACEL-17-e12720-s001.docx]

**ATF3 represses PINK1 gene transcription in lung epithelia to control mitochondrial homeostasis**

Marta Bueno^1,2^, Judith Brands^1,2^, Lauren Voltz^1^, Kaitlin Fiedler^1^, Brenton Mays^1^, John Sembrat^3^, Rama K. Mallampalli ^2,4^, M. Rojas^3^, and Ana L Mora^1,2^

^1^ Vascular Medicine Institute, Department of Medicine, University of Pittsburgh.

^2^ Division of Pulmonary Allergy and Critical Care Medicine, University of Pittsburgh, Department of Medicine, University of Pittsburgh.

^3^ The Dorothy P. and Richard P. Simmons Center for Interstitial Lung Diseases, University of Pittsburgh.

^4^ Veterans Affairs Pittsburgh Healthcare System, Pittsburgh, Pennsylvania, USA

**SUPPORTING INFORMATION**

**SUPPORTING INFORMATION LISTING**

**Supplemental Methods.**

**Supplemental Table 1.** Demographic characteristics of lung’s patient cohort

**Supplemental Table 2.** Primers use in this work.

**Supplemental Figure S1.** Tunicamycin mediates upregulation of ER stress.

**Supplemental Figure S2.** Modulation of ATF3 in A549 cells.

**Supplemental Figure S3.** The SEAP promoter activity internal control remains unmodified under the different treatments.

**Supplemental Figure S4.** Mitochondrial homeostasis and cell viability in control plasmid transfected cells.

**Supplemental Figure S5.** Time course of bleomycin-induced lung fibrosis.

**Supplemental Figure S6.** Confirmation of ATF3 deletion of mice AECIIs.

**Supplemental Figure S7.** Conditional type II lung epithelial cells ATF3 knockout mice show protection from bleomycin-induced lung fibrosis when compared with wild-type mice.

**Supplemental Figure S8.** Conditional type II lung epithelial cells ATF3 knockout mice show protection from bleomycin-induced lung fibrosis when compared with ATF3 fl/fl mice.

**Supplemental Figure S9.** PINK1 KO mice show increased lung senescence at young age.

**Supplemental Figure S10.** Upregulation of ATF3 with aging and in IPF lungs.

**SUPPORTING INFORMATION**

**Supplemental Methods:**

**Animal genotyping**

All mice used in this study were genotyped by PCR to detect ATF3 fl/fl, tetO-cre and SFTPC-rtTA sequences. For the detection of the floxed ATF3 by PCR in genotyping, the primers used were Fwd (5’-TTC ACT GCT AAT AGC TCC TG-3’), Rev. #1 (5’-TTC ATA GCT CAG GGA ACA TCG G-3’) and Rev. #2 (5’-CAA CTC CCT CTC CTC AAG TC-3’). The size of the amplified product for each of the alleles are 189bp (ATF3 WT), 347bp (ATF3 fl/fl) and 242bp (ATF3 KO). For the detection of the TetO-cre by PCR in genotyping, the primers used were: Tg-fwd (5’-GCG GTC TGG CAG TAA AAA CTA TC-3’), Tg-rev (5’-GTG AAA CAG CAT TGC TGT CAC TT-3’), WT-fwd (5’-CTA GGC CAC AGA ATT GAA AGA TCT-3’) and WT-rev (5’-GTA GGT GGA AAT TCT AGC ATC ATC C-3’). The size of the amplified product for each of the alleles are 324bp (tetO-cre WT) and 100bp (tetO-cre WT). For the detection of the SFTPC-rtTA by PCR in genotyping, the primers used were: Tg-fwd (5’-CAA ATG TTG CTT GTC TGG TG-3’), Tg-rev (5’-GTC AGT CGA GTG CAC AGT TT-3’), WT-fwd (5’-CGC TGT GGG GCA TTT TAC TTT AG-3’) and WT-rev (5’-CAT GTC CAG ATC GAA ATC GTC-3’). The size of the amplified product for each of the alleles are 200bp (SFTPC-rtTA WT) and 450bp (SFTPC-rtTA WT).

**Chromatin immunoprecipitation (ChIP) assay**

After treating 10^7^ A549 cells with tunicamycin (1 μg/ml) or DMSO (0.02%) for 5h, cultures were trypsinized and fixed in 10 ml DMEM supplemented with 1% formaldehyde for 10 min at room temperature, and then quenched by the addition of glycine to a final concentration of 125 mM for 5 minutes. After centrifugation, the cell pellets were suspended in 5 ml of cold cell lysis buffer (50 mM HEPES pH 8, 140 mM NaCl, 1 mM EDTA, 10% glycerol, 0.5% Igepal, 0.25% Triton X-100) supplemented with EDTA-free protease and phosphatase inhibitor cocktail (Sigma) and incubated on ice for 10 min. After centrifugation, the crude nuclear pellet was suspended in 5 ml of nuclear lysis buffer (10 mM Tris-HCl pH 8, 200 mM NaCl, 1 mM EDTA, 0.5 mM EGTA) supplemented with EDTA-free protease and phosphatase inhibitor cocktail. Chromatin was sheared in 2ml of buffer (50 mM Tris-HCl pH 8, 5 mM EDTA, 0.2% SDS) at 4°C, 10 times at 30 second intervals on a Branson Microtip Sonifier 450 (BLD Inc. Garner) set at constant 20% amplitude. Finally, Triton X-100 was added to a final concentration of 1% and centrifuged a high speed.

Chromatin was incubated with 2 μg of DynaI magnetic beads pre-bound to anti-ATF3 or anti-IgG rabbit control, overnight at 4°C. Magnetic beads were precipitated in a magnetic stand and washed several times with wash buffer (50mM HEPES pH 7.6; 500 mM LiCl; 1 mM EDTA; 1% Igepal and 0.7% sodium deoxycholate) and once with TE (10 mM Tris, pH 8.0; 1 mM EDTA). Finally, DNA-protein complexes were eluted from the magnetic beads in 200 ml of elution buffer (50 mM Tris–HCl, pH 8; 10 mM EDTA; 1% SDS) at 65°C for 15 min with gentle vortexing. After centrifugation, input materials and immunoprecipitated samples were decrosslinked at 65°C overnight in elution buffer. Samples were treated with RNase A at 37°C for 2 hours and then incubated with proteinase K for 30 min at 55°C. Finally, DNA samples were clean using the QIAgen PCR purification kit. PINK1 promotor fragment was amplified using primers (5’-CGT GCT GGA GTG AAC CAC TGC AAA CCT GGC-3’, and 5’-GTA ATC CCA GCT ACT CAG GTG GCT AAG GC-3’) and the B2M promotor fragment (negative locus) was amplified using primers (5’- TGC TGT CTC CAT GTT TGA TGT ATC T -3’, and 5’- TCT CTG CTC CCC ACC TCT AGG T -3’).

**Mitochondrial functional assays**

*Mitochondrial membrane potential*. A549 in 96-well plates were washed twice with 200 μl HBSS (14025092, Gibco), incubated with 2 μM of JC1 Dye (T3168, Thermo Fisher) and 1 μM of Hoechst 33342, in HBSS for 30 min at 37°C in the dark. JC-1 stains healthy mitochondria red whereas in cells with collapsed mitochondrial membrane potential, JC-1 remains in the cytoplasm and emits green fluorescence. The fluorescence of Hoechst 33342 was used to calculate the viability of the cells using a calibration curve created by serial dilution (Gero *et al.* 2013). Cells were washed twice and covered with 100 μl HBSS for fluorescence acquisition at Ex./Em. 530/580 nm JC1 red and 485/530 nm for JC1 green, and Ex./Em. 350/461 nm for Hoechst 33342.

*Mitochondrial Mass*. A549 in 96-well plates were washed twice with 200 μl HBSS and then incubated with 2 μM MitoTracker Deep Red (M22426, Thermo Fisher) and 1 μM of Hoechst 33342, in HBSS for 30 min at 37°C in the dark. Cells were then washed twice, MitoTracker fluorescence was detected at Ex./Em. 644/665 nm in 100 μl of HBSS and normalized to cell number as described in the previous section.

*Mitochondrial reactive oxygen species (mtROS).* A549 in 96-well plates were washed twice with 200 μl HBSS and then incubated with 2 μM MitoSOX (M36008, Thermo Fisher) and 1 μM of Hoechst 33342, in HBSS for 30 min at 37°C in the dark. Cells were then washed twice, MitoSOX fluorescence was detected at Ex./Em. 510/580 nm in 100 μl of HBSS and normalized to cell number as described in above.

**Supplemental Table 1. Demographic characteristics of lung’s patient cohort**

|  | **Donor-Control**  **Young** | **Donor-Control**  **Old** | **IPF** |
| --- | --- | --- | --- |
| **Subjects** | 17 | 17 | 17 |
| **Age** | 35±8  (22 – 50) | 68±7  (62 – 86) | 66±3  (62 – 74) |
| **Gender** |  |  |  |
| ***Female*** | 8 (47%) | 8 (47%) | 8 (47%) |
| ***Male*** | 9 (53%) | 9 (53%) | 9 (53%) |

**Supplemental Table 2. Primers use in this work**

| **Species** | **Gene** | **Probe Assay** | **Company** |
| --- | --- | --- | --- |
| Human | ATF3 | Hs00231069_m1 | Life Technologies |
| Human | PINK1 | Hs.PT.56a.27157402 | IDT |
| Human | CHOP | Hs.PT.58.14610020 | IDT |
| Human | XBP1 | Hs.PT.58.1903847 | IDT |
| Human | BiP | Hs.PT.58.22715160 | IDT |
| Human | RN18S | Hs.PT.47.122532.g | IDT |
| Human | p16 | Hs00923894_m1 | Life Technologies |
| Human | p19 | Hs04189686_m1 | Life Technologies |
| Human | p21 | Hs00355782_m1 | Life Technologies |
| Human | COL1A1 | Hs.PT.56a.15517795 | IDT |
| Human | FN1 | Hs.PT.58.40005963 | IDT |
| Human | FGF2 | Hs.PT.56a.24613308 | IDT |
| Mouse | ATF3 | Mm00476033_m1 | Life Technologies |
| Mouse | PINK1 | Mm.PT.56a.23711353 | IDT |
| Mouse | RN18S | Mm.PT.45.122532.g | IDT |
| Mouse | COL1A1 | Mm.PT.47.12668954 | IDT |
| Mouse | FN1 | Mm.PT.56a.5129235 | IDT |
| Mouse | p16 | Mm.PT.51.5632963 | IDT |
| Mouse | p19 | Mm.PT.51.9881334 | IDT |
| Mouse | p21 | Mm.PT.51.17125846 | IDT |
| Mouse | TGFβ | Mm.PT.47.12668954 | IDT |
| Mouse | TNFα | Mm.PT.49a.12575861 | IDT |
| Mouse | IL10 | Mm.PT.49a.11509489.gs | IDT |
| Mouse | IL6 | Mm.PT.49a.11799101.g | IDT |
| Mouse | FGF2 | Hs.PT.56a.24613308 | IDT |
| Mouse | IL1β | Mm.PT.49a.17212823 | IDT |

**Supplemental Figure S1.** **Tunicamycin mediates upregulation of ER stress.** A549 cells show induction of UPR by increased expression of ER stress markers BiP, XBP1, CHOP (A) after tunicamycin (TM) treatment. (B) Representative immunoblot analysis of ATF3 and PINK1 protein levels at different time points after TM treatment confirmed upregulation of ATF3 and decreased PINK1. Similarly, even low concentrations of TM elevate the expression of same ER stress markers (C) in primary human pulmonary alveolar epithelial cells. Data represent mean ± SEM of four (A) and three (C) independent experiments. *p<0.01, two-away ANOVA with multiple comparison test.

**Supplemental Figure S2. Modulation of ATF3 in A549 cells.** After overexpression of ATF3 (or GFP as transfection control) for 48h, q-RTPCR was performed in those cells. Higher mRNA levels of ATF3 (A) were followed by lover expression of PINK1 (B) and upregulation of several ER stress marker (BiP, CHOP, XBP1 in panel C). In addition, (D) overexpression of ATF3 in A549 cells resulted in increased levels of some fibrotic markers (COL1A1 and FN1) but not others such as FGF2. (E) Also, a substantial induction of the senescence marker p21 was found in ATF3-overexpressing cells. (F) Representative immunoblot at 48h of ATF3 protein levels after TM treatment in the presence or absence of *ATF3* silencing Data are reported as mean ± SEM of four independent experiments. *p<0.01, unpaired, 2-tailed Student’s t test vs. GFP transfected sample.

**Supplemental Figure S3. The SEAP promoter activity internal control remains unmodified under the different treatments.** (A) A549 cells were treated with a low concentrations of DMSO (vehicle) or TM (1 μg/ml) for 5h. Chromatin immunoprecipitation (ChIP) assays on the B2M promoter (negative locus) were performed using antibodies against ATF3 and an IgG isotype control. (B) A549 cells transfected with human *PINK1* promoter luciferase reporter (containing secreted alkaline phosphatase –SEAP- as internal control) were treated with or without 10 μg/ml of TM. SEAP activities were measured after 24 h stimulation. SEAP (secreted alkaline phosphatase) activities were measured after 24 h stimulation. (C) PINK1 and (D) SEAP activities were measures for A549 cells transfected with human PINK1 promoter luciferase reporter and co-transfected with ATF3 or GFP overexpressing plasmids. Luciferase activities were normalized to SEAP activities. Values obtained for the un-treated (0 μg/ml TM) or no co-transfected (NT) sample represent 100%. Data are reported as mean ± SEM of four independent experiments.

**Supplemental Figure S4. Mitochondrial homeostasis and cell viability in control plasmid transfected cells.** A549 cell were transfected with increased quantities of V5-tag empty plasmid for 48h, and cell viability and mitochondrial health were measured. No changes in cell viability (A), mitochondrial mass (B), depolarization (C) or mitochondrial ROS production (D) were detected. Data represent mean ± SEM of 24 replicates per condition, in three independent experiments.

**Supplemental Figure S5.** **Time course of bleomycin-induced lung fibrosis.** Fibrotic markers, COL1A1 (A) and FN1 (B), continuously increases during the 15 days after bleomycin instillation. (C) Higher collagen deposition in bleomycin-treated wild-type mice was confirmed by hydroxyproline assay. (D) Differential cells populations in the BAL (bronchoalveolar lavage) fluid at 0, 1, 7 and 15 days after bleomycin. Data represent mean ± SEM (*n*=6). *p<0.01 vs. day 0, one-away ANOVA with multiple comparison test.

**Supplemental Figure S6.** **Confirmation of ATF3 deletion of mice AECIIs.** (A) Using a Cre-tdTomato reporter mice bred with our tet-o-Cre/SPC-rtta mice we confirmed location of the Cre recombinase expression (dTomato) in lung type II epithelial cells (SPC) after four week of doxycycline hyclate treatment to promote recombination. (B) Verification of recombination only in the lung (not the liver) in mice receiving doxycycline diet for 4 weeks. (C) Total lung lysate from conditional type II lung epithelial cells ATF3 knockout mice show less expression of *ATF3* mRNA. Data are reported as mean ± SEM (*n*=8). *p<0.01, unpaired, 2-tailed Student’s t test vs. ATF3 fl/fl.

**Supplemental Figure S7.** **Conditional type II lung epithelial cells ATF3 knockout mice show protection from bleomycin-induced lung fibrosis when compared with wild-type mice.** (A) Even in the areas where the injury is more severe, the ATF3 spc-KO mice has less collagen deposition (in blue, by Masson trichrome staining). After 15 days post bleomycin instillation, mRNA levels of FG2 (B) and IL1β (C) are reduced in ATF3 spc-KO mice. (D) Differential cells populations in the BAL (bronchoalveolar lavage) fluid at 15 days after bleomycin showing less inflammation in the ATF3 spc-KO mice. Data represent mean ± SEM (*n*=6-8). *p<0.01 vs. ATF3 WT PBS and ^#^p<0.01 as indicated, two-away ANOVA with multiple comparison test.

**Supplemental Figure S8.** **Conditional type II lung epithelial cells ATF3 knockout mice show protection from bleomycin-induced lung fibrosis when compared with ATF3 fl/fl/ mice.** (A) Biochemistry determination of collagen deposition by hydroxyproline assay at day 15 after bleomycin. Transcript mRNA levels of ATF3 (B) and PINK1 (C) at day 15 after bleomycin. Other fibrotic (D–G) and inflammatory markers (H-K). (L) Differential cells populations in the BAL (bronchoalveolar lavage) fluid at 15 days after bleomycin showing less inflammation in the ATF3 spc-KO mice. (M) ATF3 spc-KO mice present lower levels of senescence markers in total lung lysate. Data are reported as mean ± SEM (*n*=8). *p<0.01, unpaired, 2-tailed Student’s t test vs. ATF3 fl/fl.

**Supplemental Figure S9.** **PINK1 KO mice show increased lung senescence at young age.** PINK1 KO mice show higher transcript levels of p16 (A), p19 (B) and p21(C) that their wild-type littermate controls. Data represent mean ± SEM (*n*=5). *p<0.01 vs. 3mo and ^#^p<0.01 as indicated, two-away ANOVA with multiple comparison test.

**Supplemental Figure S10. Upregulation of ATF3 with aging and in IPF lungs.** (A) Representative immunoblot analysis of ATF3 protein expression in total lung lysates of young donor (less than 50 years old), old donor (more than 50years old) and IPF lungs. (B) Single channel signal of representative immunoflourescence using anti–ABCA3 (type II epithelial cell marker; red) and anti-ATF3 (green) antibodies plus DAPI to stain nuclei blue, showing high ATF3 expression in the nuclei of hyperplasic AECIIs from honeycombs in IPF lung.

**Supplemental Figure S1**


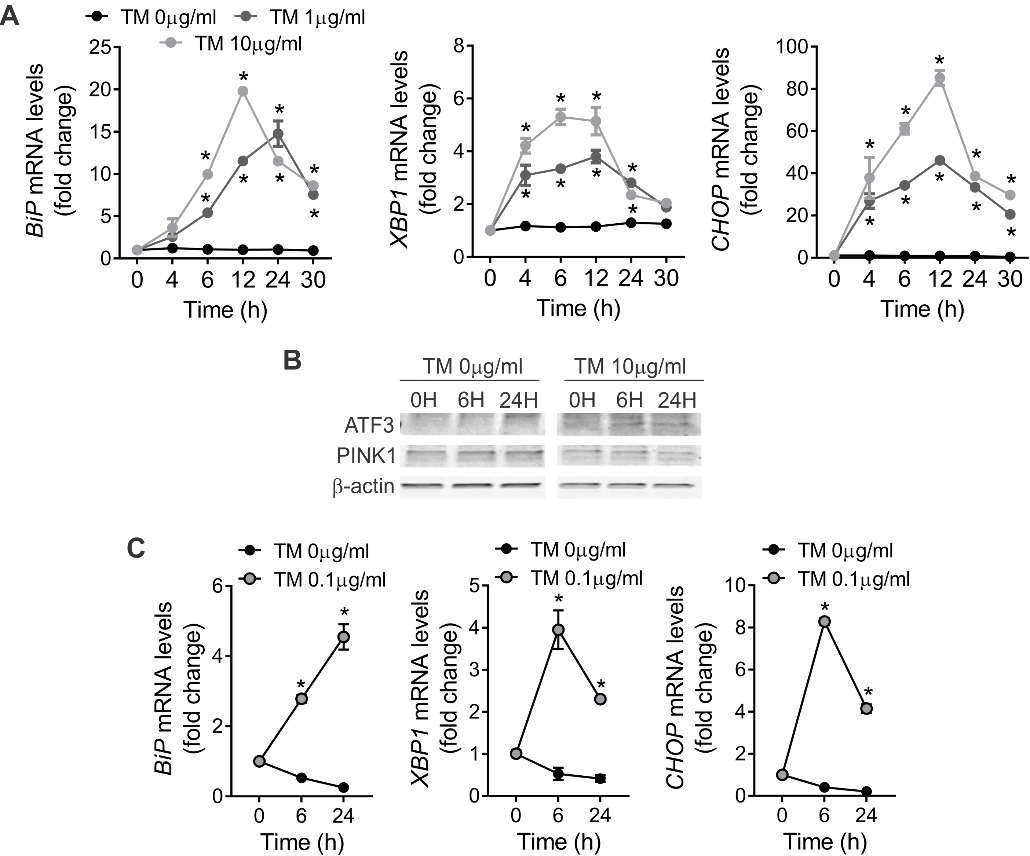


**Supplemental Figure S2**

**
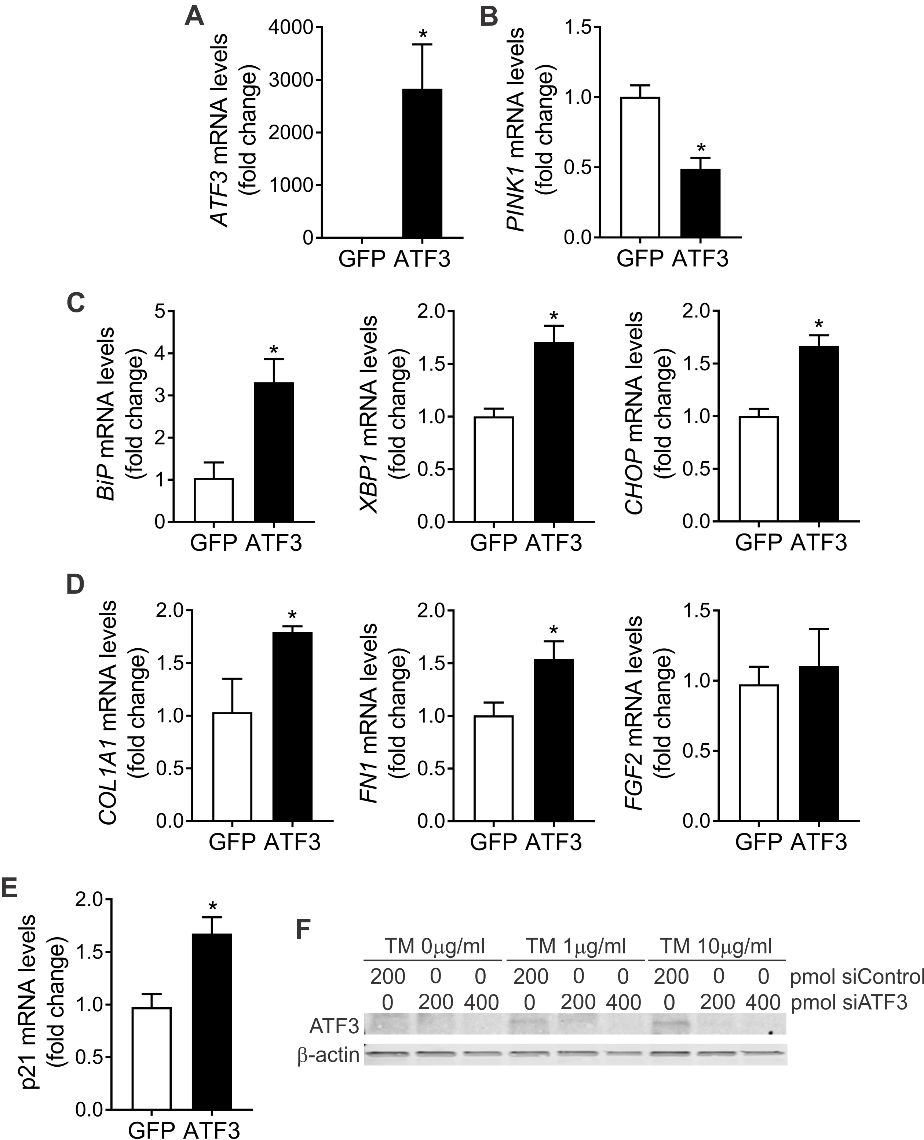
**

**Supplemental Figure S3**

**
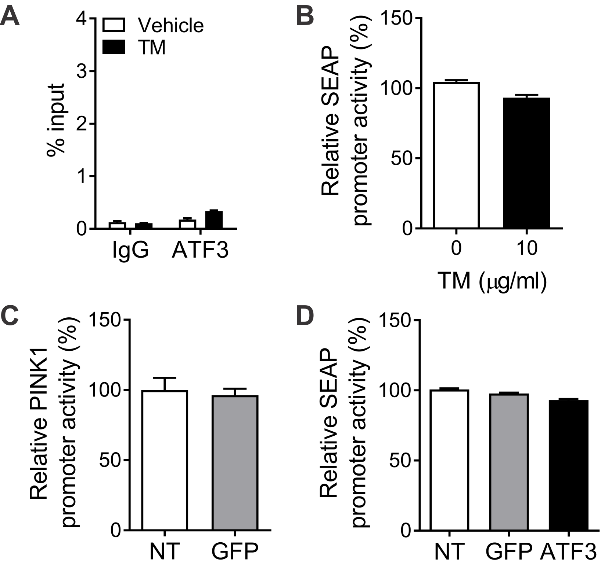
**

**Supplemental Figure S4**

**
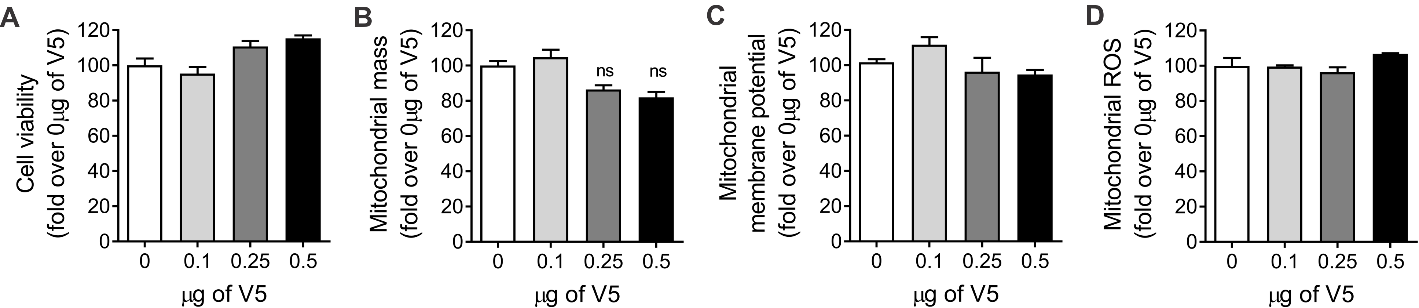
**

**Supplemental Figure S5**

**
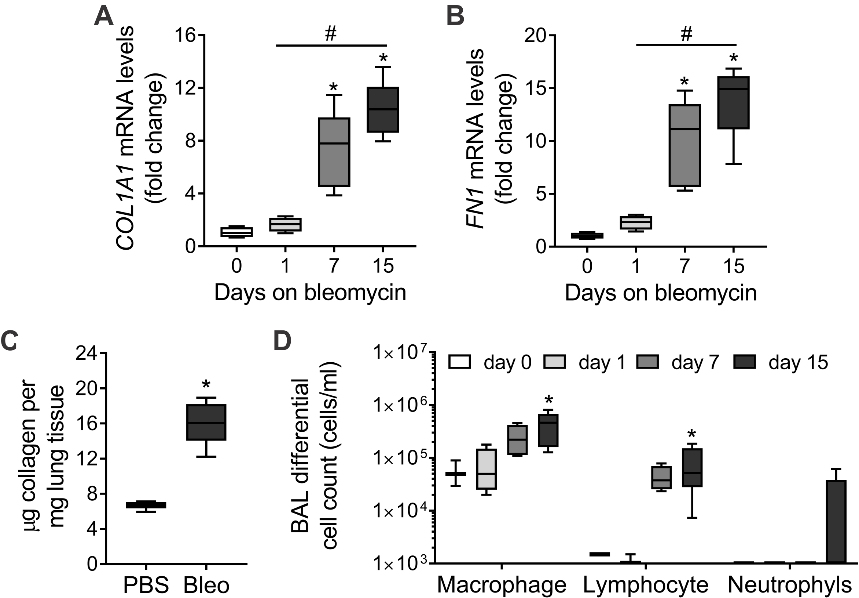
**

**Supplemental Figure S6**

**
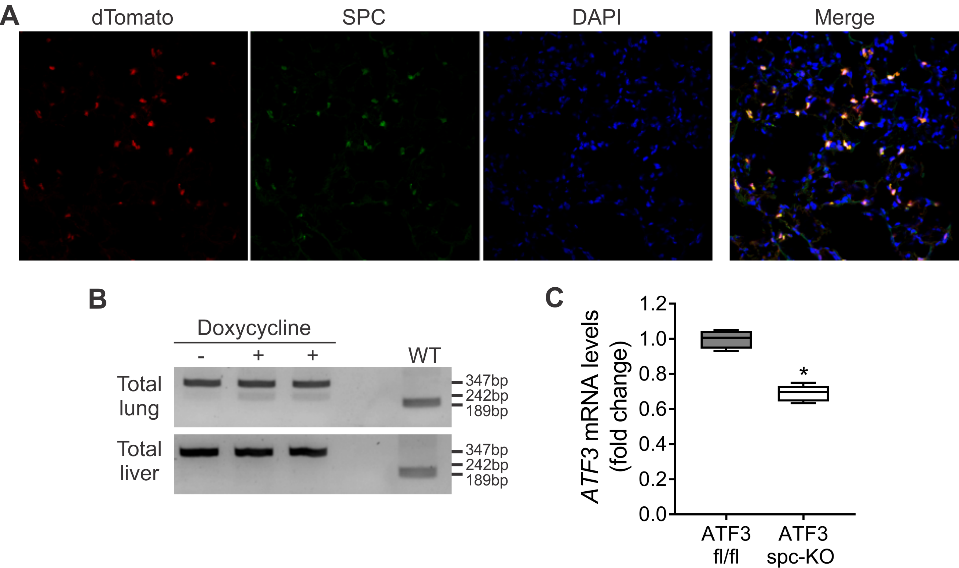
**

**Supplemental Figure S7**

**
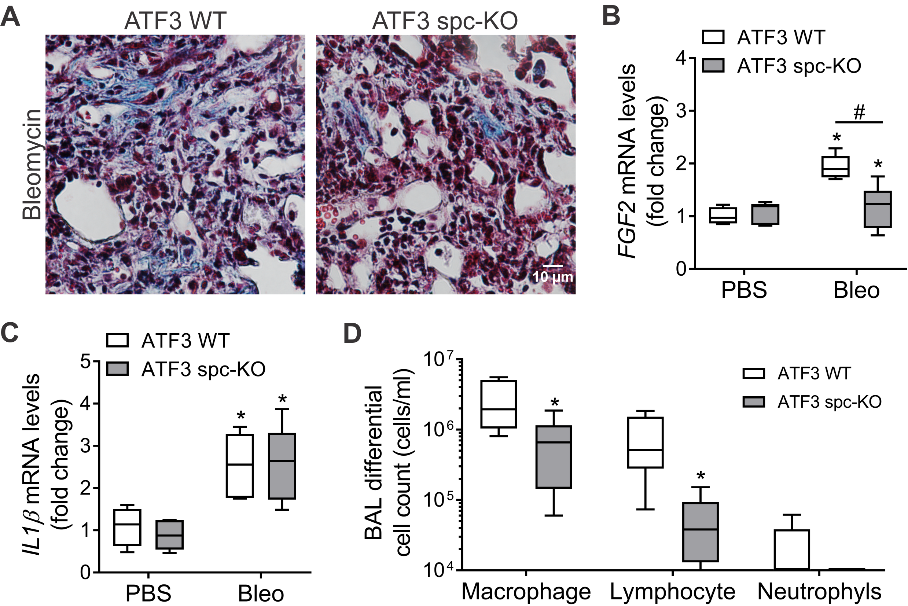
**

**Supplemental Figure S8**

**
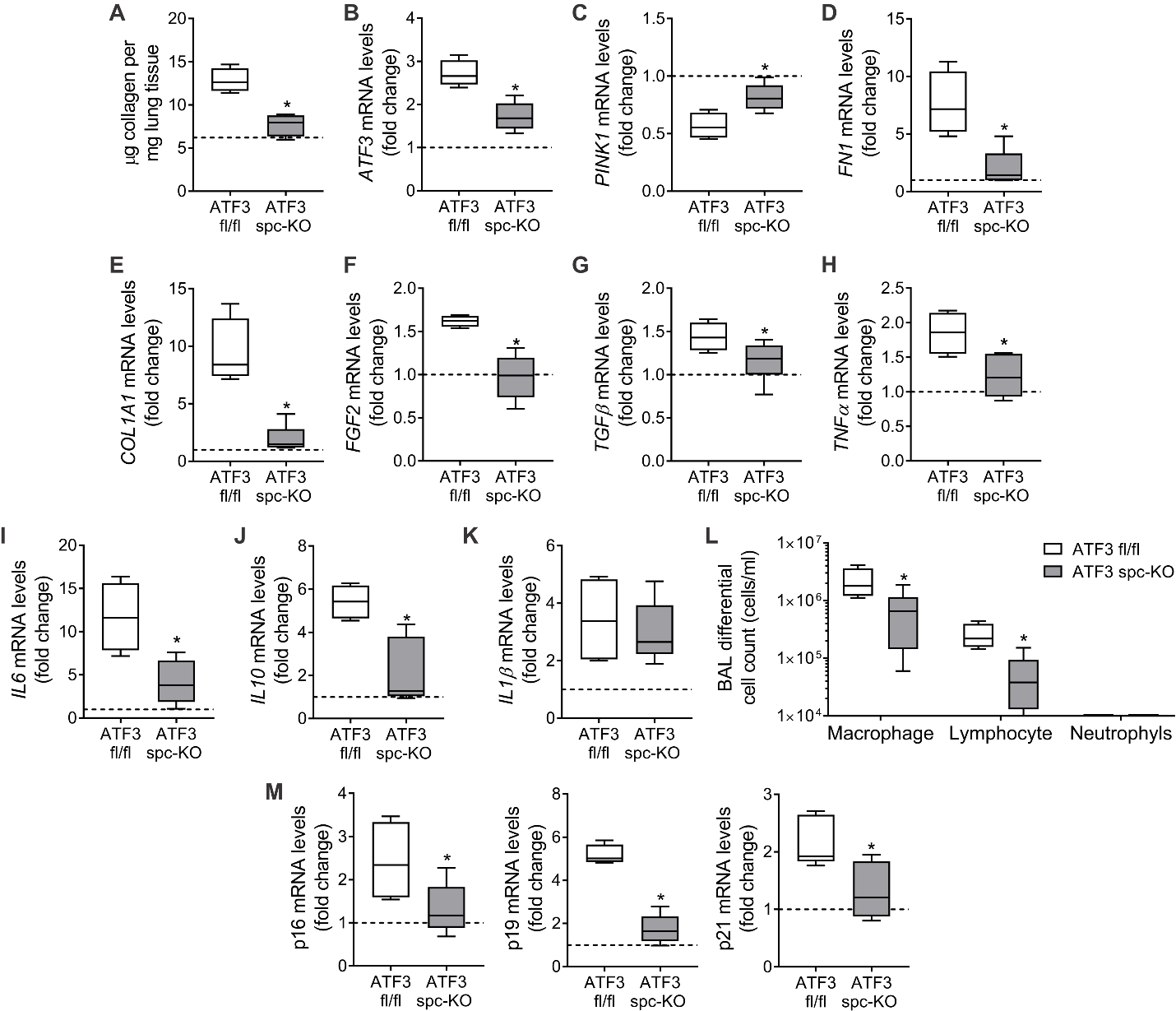
**

**Supplemental Figure S9**

**
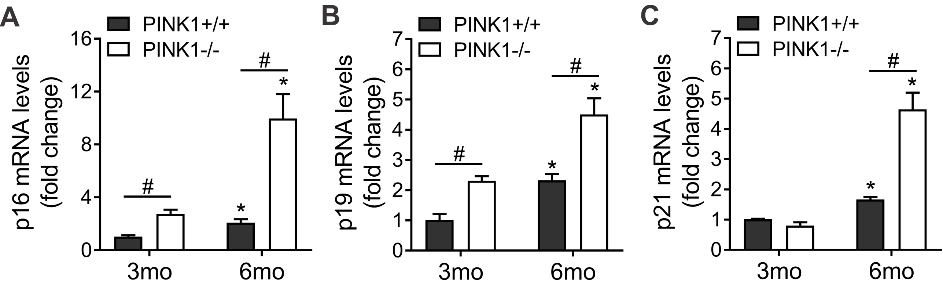
**

**Supplemental Figure S10**

**
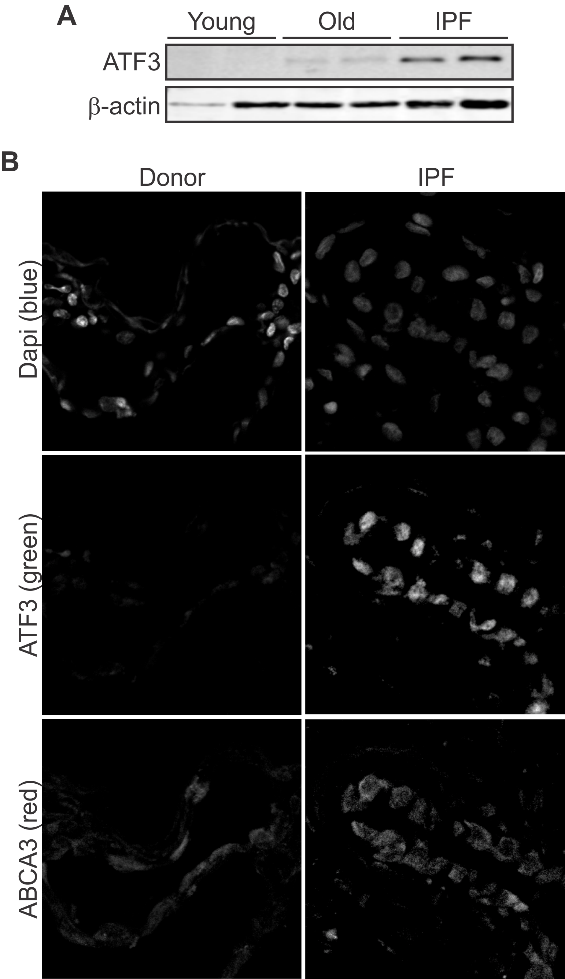
**
